# Dental implants and diabetes mellitus—a systematic review

**DOI:** 10.1186/s40729-016-0038-2

**Published:** 2016-02-11

**Authors:** Hendrik Naujokat, Burkhard Kunzendorf, Jörg Wiltfang

**Affiliations:** Klinik für Mund- Kiefer- und Gesichtschirurgie, Universitätsklinikum Schleswig-Holstein, Campus Kiel, Arnold-Heller-Straße 3, 24105 Kiel, Germany

**Keywords:** Dental implants, Implant survival, Diabetes mellitus, Glycemic control, Peri-implantitis, Systemic disease, Risk factor

## Abstract

Dental implant surgery has developed to a widely used procedure for dental rehabilitation and is a secure and predictable procedure. Local and systemic risk factors can result in higher failure rates. Diabetes mellitus is a chronic disease that goes in with hyperglycemia and causes multifarious side effects. Diabetes as a relative contraindication for implant surgery is controversially discussed. Because the number of patients suffering from diabetes increases, there are more diabetic patients demanding implant procedures. We aimed to answer the PICO question “Do diabetic patients with dental implants have a higher complication rate in comparison to healthy controls?” by a systematic literature search based on the PRISMA statement. We identified 22 clinical studies and 20 publications of aggregated literature, which were quite heterogeneous concerning methods and results. We conclude that patients with poorly controlled diabetes suffer from impaired osseointegration, elevated risk of peri-implantitis, and higher level of implant failure. The influence of duration of the disease is not fully clear. The supportive administration of antibiotics and chlorhexidine seems to improve implant success. When diabetes is under well control, implant procedures are safe and predictable with a complication rate similar to that of healthy patients.

## Review

### Introduction

Today, dental implants are one of the restorative methods to replace missing teeth. Improvements in implant design, surface characteristics, and surgical protocols made implants a secure and highly predictable procedure with a mean survival rate of 94.6 % and a mean success rate of 89.7 % after more than 10 years [1]. Implant survival is initially dependent on successful osseointegration following placement. Any alteration of this biological process may adversely affect treatment outcome. Subsequently, as an implant is restored and placed into function, bone remodeling becomes a critical aspect of implant survival in responding to the functional demands placed on the implant restoration and supporting bone. The critical dependence on bone metabolism for implant survival leads us to evaluation of certain risk factors. One of the controversial discussed diseases is diabetes mellitus. Diabetes mellitus is a chronic metabolic disorder that leads to hyperglycemia, which raises multiple complications caused by micro- and macroangiopathy. Diabetic patients have increased frequency of periodontitis and tooth loss [2], delayed wound healing [3], and impaired response to infection. In 1980, more than 150 million people worldwide were affected and that number had grown to 350 million by 2008 [4]. This trend highlights the need for better understanding of diabetes and its therapy and its impact on dental implant rehabilitation. In the past, diabetes was long time seen as a relative risk factor to dental implants. In contrast, today, there is a change in paradigm. Recent studies offer indirect evidence for diabetes patients benefiting from oral rehabilitation based on dental implant therapy. After tooth loss, patients avoid food which needs more effort to masticate which can lead to an adverse nutrition with poor metabolic control. A sufficient dental rehabilitation allows the patient to improve nutrition and the metabolic control. On the other hand, it is still unclear how quality of diabetes therapy and duration of disease influence the success of dental implants. The ability to anticipate outcomes is an essential part of risk management in dental implant surgery. Recognizing conditions that place the patient at a higher risk of complications will allow the surgeon to make informed decisions and refine the treatment plan to optimize the outcomes [5].

Therefore, we conducted a systematic review of published clinical studies to investigate whether dental implant placement in diabetic vs. non-diabetic patients yields any detrimental effects on postoperative complications, peri-implantitis, and implant failure rate. The main goal is to get a more detailed view on the influence of quality of glycemic control and duration of disease to give recommendation for treatment options and surgical protocols.

### Materials and methods

The substructure of the systematic review is based on the PRISMA statement [6]. The focused question according to the PICO schema is: “Do diabetic patients with dental implants have a higher complication rate in comparison to healthy controls?”

#### Search strategies

The systematic literature search was performed by an independent scientist (Burkhard Kunzendorf). The following databases were incorporated in the systematic search for relevant literature: PubMed, Embase, AWMF Online, National Guideline Clearinghouse, Guidelines International Network, and Cochrane Library. The following search terms were used: dental implants AND diabetes, transgingival implants AND diabetes, maxillary augmentation AND diabetes, mandibular augmentation AND diabetes, peri-implantitis AND diabetes, Zahnimplantate AND Diabetes, Kieferkammaufbau AND Diabetes, Periimplantitis AND Diabetes. Electronic search was complemented by an iterative hand-search in the reference lists of the already identified articles. The time period of the literature search was between 10 April and 7 May 2015. Endnote X7 was used for the electronic management of the literature.

#### Study inclusion and exclusion criteria

During the first stage of study selection, the titles and abstracts were screened and evaluated according to the following inclusion criteria: English or German language, retrospective and prospective clinical trials, observational studies, cross-sectional studies, cohort studies, and case series. During this procedure, the pre-selected publications were evaluated according to the following exclusion criteria: in vitro studies, animal studies, case reports with less than 10 patients, and publications older than 15 years.

#### Quality and risk of bias assessment of selected studies

A quality assessment of all selected full-text articles was performed. It made no sense to use the Cochrane collaboration tool for assessing risk of bias for randomized controlled studies since the majority of the included studies were not randomized or retrospective case series. Instead, a system modified from the US Agency for Healthcare Research and Quality Methods Guide for Comparative Effectiveness Reviews was used, which asked for the sources of possible bias [7]. The criteria were each judged with low, medium, high, or unknown risk of bias: case selection bias and confounding, attrition bias (loss of participants), detection bias (reliable measures), and reporting bias (selective or incomplete reporting), followed by a summary of the risk.

### Results

#### Study selection

**Fig. 1:**
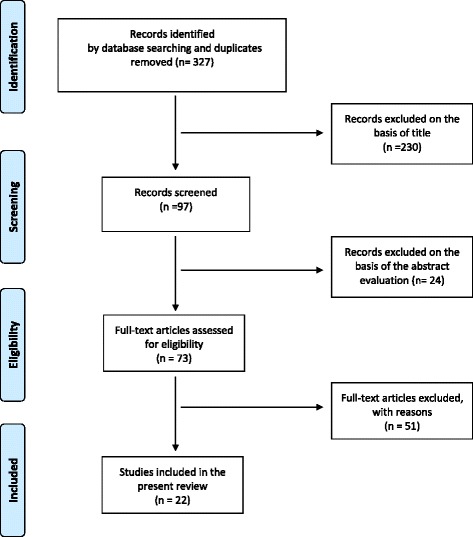
Selection process of the included literature

There are no guidelines existing to the topic of dental implants and diabetes mellitus. A total of 327 potentially relevant titles and abstracts were found by the electronic search and additional evaluation of reference lists. During the first screening, 230 publications were excluded based on the title and keywords. Additionally, 24 titles were excluded based on abstract evaluation. Seventy-three full-text articles were thoroughly evaluated. A total of 51 papers had to be excluded at this stage because they did not fulfil the inclusion criteria of the present systematic review. Twenty-two articles went into qualitative assessment (Fig. 1). Because of too few studies, heterogenic study design, and incompletely reported data like type of diabetes therapy, quality of glycemic control, and duration of disease, the quantitative data synthesis could not be performed in the way necessary for meta-analysis. Additionally, we identified 20 reviews and meta-analyses. They are excluded from our results.

#### Evaluation of study quality and risk of bias

The majority (*n* = 12) of the 22 studies were prospective and one of these studies was a multicenter study. Eight were retrospective and two were cross-sectional studies. Randomization of patients was not readable in any of the studies (Table 1). Despite a low evidence level in terms of study design, there were no major concerns about risk of bias; 13/22 studies were rated with a low risk of bias, 9/22 had a medium risk. No study had a high risk of bias, and consequently no further study was excluded at this stage because of bias (Table 2).

**Table 1:** List of the included studies and its main characteristics

Author	Year	Study type	Diabetes type	Control	Diabetes therapy	Glycemic control [HbA1c %]	Duration of diabetes (years)	Number of patients	Number of implants	Duration of study (years)	Implant survival [%]	Conclusion
Alsaadi	2007	Retrospective	Type II	Non-diabetes	n.d.	n.d.	n.d.	2004 (overall)	6946 (overall)	6 months	96.4 (global)	Diabetes does not cause higher failure rate in the first 6 months.
Aguilar-Salvatierra	2015	Prospective	Type II	3 groups (HbA1c)	n.d.	6–8 (well), 8–10 (moderately), >10 (poorly)	n.d.	85	85	2	100 vs. 96.6 vs. 86.3	Patients with diabetes can receive implant-based treatments, providing they present moderate HbA1c values. Peri-implantitis increases with elevated HbA1c.
Anner	2010	Retrospective	n.d.	Non-diabetes	n.d.	n.d.	n.d.	49 diabetes, 475 overall	1626	3 ± 2	97.2 vs. 95	Diabetes was not related to implant survival in this patient cohort.
Busenlechner	2014	Retrospective	n.d.	Non-diabetes	n.d.	n.d.	n.d.	4316	>10,000	8 years	95.1 vs. 97	Diabetes does not have any influence on implant survival after 8 years, if blood sugar is effectively controlled.
Daubert	2015	Cross-sectional	n.d.	Non-diabetes	n.d.	n.d.	n.d.	8 diabetes, 96 overall	225	10	n.d.	Significant associations between implant failure and diabetes (relative risk 4.8 and 3.3) and peri-implant diseases and diabetes (relative risk 4.1).
Dowell	2007	Prospective	Type II	Non-diabetes	Diet, oral, insulin and combination	6–8 (well), 8–10 (moderately), >10 (poorly)	n.d.	25 diabetes, 10 non-diabetes	38 diabetes, 12 non-diabetes	4 months	100	Diabetes has no negative influence; the quality of glycemic control has no effect on implant success.
Erdogan	2014	Prospective	Type II	Non-diabetes	n.d.	Mean 6.8	7.5	12 diabetes, 12 control	43	1	100	No significant difference for wound healing, radiographic findings, implant success and volume of augmentation (guided bone regeneration with bone scrapes and bone substitute material).
Ferreira	2006	Cross-sectional	n.d.	Non-diabetes	n.d.	Blood sugar >126 mg/dl or diabetic medication subscribed	n.d.	212 (overall)	578 (overall)	6 months–5 years	n.d.	Risk for peri-implantitis in “uncontrolled” diabetes is 1.9 times higher compared to the non-diabetes group.
Fiorellini	2000	Retrospective	Types I and II	None	n.d.	“Proper levels of glycemic control”	8.9 ± 14.3	40	215	6.5	85.6	Survival rate is lower than for general population, but there is still a reasonable success rate. Most implant failures are in the first year after loading.
Ghiraldini	2015	Prospective	Type II	Non-diabetes	n.d.	<8 (better) >8 (poorly)	10.7 ± 5	16 better, 16 poorly, 19 control	51	1	100	Poor glycemic control negatively modulated the bone factors during healing, although diabetes (regardless of glycemic control) had no effect on implant stabilization.
Gomez-Moreno	2014	Prospective	Type II	4 groups (HbA1c)	n.d.	<6 (healthy), 6–8 (well), 8–10 (moderately) >10 (poorly)	n.d.	67	67	3	n.d.	Elevated HbA1c causes more bone loss (not significant) and significantly higher BOP. Probing depth is not influenced by glycemic control.
Khandelwal	2011	Prospective	Type II	2 different types of implants	n.d.	7.5–11.4 (poorly controlled)	n.d.	24	48	4 months	98	Successful implant therapy in patients suffering poorly controlled diabetes. No difference between the two implant systems.
Morris	2005	Prospective	Type II	Non-diabetes	n.d.	n.d.	n.d.	663	255 diabetes, 2632 non-diabetes	3	92.2 and 93.2, respectively	Diabetic patients tend to have more failures than non-diabetic patients. The use of CHX resulted in a slight improvement in survival in non-diabetic patients and in a greater improvement in type II patients, the same effect for antibiotic use.
Moy	2005	Retrospective	n.d.	Non-diabetes	n.d.	n.d.	n.d.	48 diabetes, 1140 overall	4684 (overall)	up to 20	n.d.	Significantly increased relative risk for implant failure (relative risk = 2.75).
Oates	2009	Prospective	Type II	Non-diabetes	Diet, oral, insulin and combination	6–8 (well), 8–10 (moderately), >10 (poorly)	n.d.	32	42	4 months		Patients with poorly controlled HbA1c have lower stability in the first 2–6 weeks, but it reaches the baseline in the following weeks. But reaching the baseline takes two times the duration it needs in the non-diabetic group.
Oates	2014	Prospective	Type II	Non-diabetes	n.d.	6–8 (well), >8 (poorly)	n.d.	44 well, 19 poorly, 49 control	220	1	99	The initial implant stability is lower in diabetic patient, but 1 year after insertion there in so difference even in the poorly controlled group. Diabetes has no influence on implant survival.
Olson	2000	Prospective, multicenter	Type II	None	Diet, oral, insulin and combination	n.d.	n.d.	89	178	5	91 vs. 88	Implants in mandibular symphysis in diabetic patient are a predictable procedure. Duration of diabetes may be associated with implant failure, CHX improves implant survival.
Peled	2003	Retrospective	Type II	None	n.d.	“Well-controlled,” no data for HbA1c	n.d.	41	141	1 and 5	97.3 vs. 94.4	No correlation was found between failed implants and glucose level. The clinical outcome of dental implants in a selected group of patients with well-controlled type II diabetes mellitus is satisfying and encouraging.
Tawil	2008	Prospective	Type II	Non-diabetes	n.d.	<7 (well), 7–9 (moderately), >9 (poorly) mean 7.2	n.d.	54 diabetes, 54 control	255 diabetes, 244 control	1 to 12	97.2 vs. 98.8	No significant difference for implant survival between the groups and no difference between good and medium glycemic control for bone resorption. Augmentations caused no complications. Duration of diabetes was no confounder.
Tatarakis	2013	Prospective	Type II	None	n.d.	Mean 7.1	n.d.	32	>32	1	n.d.	The clinical, microbiological, salivary biomarkers and psychosocial profiles of patient with diabetes under good control are very similar to those of non-diabetes.
Turkyilmaz	2010	Retrospective	Type II	None	Diet, oral, insulin and combination	5–10	5–21	10	23	1	100	No evidence of diminished clinical success, BOP negative, no pathological probing depth, marginal bone loss 0.3 ± 0.2 mm.
Zupnik	2011	Retrospective	n.d.	Non-diabetes	n.d.	n.d.	n.d.	n.d.	25 diabetes, 316 non-diabetes	4	96.4 (global)	Implant failure (explantation) is 2.57 times higher for patient with diabetes than patients without diabetes after 4 years.

*n.d.* no data provided

**Table 2:** Risk of bias of the included studies

Author	Year	Study type	Selection bias (homogeneity and confounders)	Performance bias (fidelity to protocol)	Attrition bias (loss of participants)	Detection bias (reliable measures)	Reporting bias (selective reporting or conflict interests)	Summary assessment risk of bias
Alsaadi	2007	Retrospective	H	U	U	L	L	L
Aguilar-Salvatierra	2015	Prospective	H	L	L	L	L	L
Anner	2010	Retrospective	H	U	U	L	L	M
Busenlechner	2014	Retrospective	H	L	U	L	L	L
Daubert	2015	Cross-sectional	H	L	U	L	L	L
Dowell	2007	Prospective	H	L	L	L	M	M
Erdogan	2014	Prospective	H	L	L	L	M	M
Ferreira	2006	Cross-sectional	H	L	L	L	L	L
Fiorellini	2000	Retrospective	H	M	U	M	L	M
Ghiraldini	2015	Prospective	H	L	L	L	L	L
Gomez-Moreno	2014	Prospective	H	L	L	L	L	L
Khandelwal	2011	Prospective	L	L	L	M	L	L
Morris	2005	Prospective	H	L	L	M	L	M
Moy	2005	Retrospective	H	L	U	M	L	M
Oates	2009	Prospective	H	L	L	L	L	L
Oates	2014	Prospective	H	L	M	L	L	L
Olson	2000	Prospective, multicenter	H	L	U	L	L	L
Peled	2003	Retrospective	H	L	U	M	L	M
Tawil	2008	Prospective	H	L	L	M	L	M
Tatarakis	2013	Prospective	H	L	L	L	L	L
Turkyilmaz	2010	Retrospective	H	L	U	L	L	L
Zupnik	2011	Retrospective	H	L	U	M	L	M

*L* low, *M* medium, *H* high, *U* unknown risk of bias

#### Diabetes and osseointegration

Osseointegration describes the process of formation of a direct interface between the implant and bone, without intervening soft tissue. This process is prerequisite for implant stability and inflammation-free survival. It includes remodeling of the surrounding bone with migrations and proliferation of osteoblasts and supporting connective tissue. We identified two prospective studies investigating the influence of type II diabetes on osseointegration. They are published by the same author but are independent studies from different years [8, 9]. In both studies, the patients included were stratified by HbA1c levels as well-controlled (HbA1c 6.1–8 %), moderately controlled (HbA1c 8.1–10 %), and poorly controlled (HbA1c ≥10 %). The healthy control had HbA1c ≤6 %. Patients with poorly controlled diabetes have lower stability at the first 2 to 6 weeks. In the following weeks, stability reaches the baseline again, but reaching baseline takes two times the duration it needs in the healthy treatment group. Looking at the implant stability 1 year after implantation, there is no difference between the groups, not even to the poorly controlled HbA1c.

#### Diabetes and peri-implantitis

We found two prospective, two cross-sectional, and one retrospective study examining the influence of diabetes on peri-implantitis. The conclusions are quite heterogeneous. The study of Aguilar-Salvatierra [10] started to evaluate 2 years after insertion and found that the number of patients suffering from peri-implant inflammation increases with elevated HbA1c values. The population was divided into well-controlled (HbA1c 6–8 %), moderately controlled (HbA1c 8–10 %), and poorly controlled (HbA1c >10 %), but there was no healthy control. The two cross-sectional studies yield an elevated relative risk for peri-implantitis of 1.9 and 4.1 caused by diabetes [11, 12]. The duration of these studies was 6 months to 5 years and 10 years, respectively. On the other hand, the retrospective study of Turkyilmaz [13] showed no evidence of diminished clinical success 1 year after implantation, defined by negative bleeding on probing, no pathological probing depth, and a marginal bone loss of 0.3 ± 0.1 mm in a population of type II diabetics. The results in the prospective study of Gomez-Moreno [14] show that elevated HbA1c causes more bone resorption after 3 years, but this effect is not significant. The bleeding on probing is more often in the poorly controlled population, but the probing depth is not increased.

#### Diabetes and implant survival

Implant survival is an easily defined and measured endpoint for dental implant therapy. Nearly every study reports its implant survival rate. Our literature search identified 18 publications with these data. We divided them into two groups: the first one covers 7 studies with observation time up to 1 year (6 prospective, 1 retrospective studies), the second one longer periods (4 prospective, 1 cross-sectional, and 6 retrospective studies). In the short-time group, 5 of the studies had a healthy control group. The result for implant survival in diabetics is 100 to 96.4 %, which does not differ from the healthy control [9, 15–18]. The 2 studies without control group report a 100 % survival rate 4 months and 1 year after implantation [13, 19]. The time periods in the long-time group differ from 1 year up to 20 years and are very heterogeneous. We found 4 prospective, 6 retrospective, and 1 cross-sectional study. Seven studies compared the diabetic survival rates to healthy population, and results are equivocal. On the one hand, survival rates of diabetics are similar to healthy control: 95.1 vs. 97 %, 97.2 vs. 95 %, 92 vs. 93.2 %, and 97 vs. 98.8 % [20–23]. On the other hand, there are 2 studies reporting relative risk for implant failure in diabetic patients elevated to 4.8 and 2.75, respectively [11, 24]. The studies without a healthy control present survival rates from 100 to 86 % [10], 97.3 and 94.4 % after 1 and 5 years [25], and 91 to 88 % after 5 years [26], which are comparable to survival rates in healthy individuals. There is one work that demonstrates survival rate of 85.6 % after 6 years, which is lower than that in healthy population. The most implant failures were observed in the first year after prosthetic loading [27].

#### Diabetes and bone augmentation

We identified two prospective studies that evaluated “advanced” implant surgery covering sinus lift procedure and guided bone regeneration. The study of Erdogan consists of type II diabetics moderately and well-controlled (HbA1c 6–7.5 %) with a mean duration of disease of 7.5 years and a healthy control group. Augmentation of the maxilla was performed by guided bone regeneration with autologous bone from the mandibular ramus harvested by bone scrapers, a synthetic bone substitute, and collagen membrane. The result after 1 year is that patients with HbA1c levels <7.5 % may undergo staged guided bone regeneration securely [16]. The other study consisted of a larger group of type II diabetic patients and healthy control which were treated with simple or advanced implant therapy. The authors conclude that well- to fairly well-controlled diabetic patients with a mean HbA1c of 7.2 % had the same overall survival rate as controls in conventional and advanced implant therapy. No difference was seen when looking at bone resorption [23].

#### Influence of quality of glycemic control

When looking at the question, if diabetes is a risk factor for dental implants, it is not sufficient to decide having diabetes or not. The greater the impact of diabetes, the worse the patient handles with glycemic control. In international studies, the percentage of glycosylated hemoglobin is an indicator for glycemic levels from previous 6–8 weeks. Unfortunately, many studies do not provide data of HbA1c. Some authors call their patient “under well control” or “poorly controlled,” without representing any definition. Our search identified seven studies with a clear definition of different qualities of glycemic control by HbA1c. Three defined HbA1c 6–8 % as good, 8–10 % as moderately, and >10 % as poorly controlled. Two studies called HbA1c <8 % better and >8 % poorly controlled and another <7 % well, 7–9 % moderately, and >9 % poorly controlled. While four studies conclude better implant survival and less peri-implant complications in the well-controlled group [8, 10, 14, 17], the three others see no difference in implant success even in the poorly controlled patients [9, 15, 23]. The study of Khandelwal treated exclusively patients with poor glycemic control (HbA1c 7.5–11.4 %) and had 98 % implant survival, after 4 months; therefore, he concluded that implant therapy is successful even in poorly controlled diabetes [19].

#### Influence of duration of diabetes disease

It is plausible that with extended duration of diabetes, the systemic side effects increase. However, the influence of duration of the disease on implant surgery outcome is only very little examined. Most of the included studies (17 of 22) provided no data about duration since diagnosis of diabetes. In five studies, these data were given, but only two of them analyzed the influence on the implant survival. While Olsen concludes that the duration of diabetes may be associated with implant failure [26], Tawil says that implant survival is independent from diabetes duration [23].

#### Influence of supportive therapy

Although there is some controversy over the use of antibiotics in healthy patients, these are recommended in diabetic patients in implant surgery. The reason is the impaired immune system, which can lead to wound infections and healing complications. Some authors indicate the administration of antibiotics for 5–7 days postoperatively; others support the view that there is no significant reduction of wound infection when using antibiotics more than 1 day after surgery. Our literature search resulted in one prospective study that shows a clear benefit using preoperative antibiotics in both type II diabetics and non-diabetics. For implants in the non-type II diabetic group, survival for those implants placed with preoperative antibiotics was 4.5 % higher than implants not provided coverage at placement surgery. This improvement in survival was even greater (10.5 %) for those in the type II diabetic group. These outcomes are clinically important and should be considered clinically significant [22]. But the authors do not describe which antibiotic they used and how long it was administered.

There was a large improvement in implant survival in the type II diabetic patients when chlorhexidine (CHX) (95.6 %, 4.4 % failures) was used at the time of implant placement, as compared to when CHX was not used (86.5 %, 13.5 % failures). This difference in survival (9.1 %) was large enough to be considered clinically significant but was not found in the non-type II diabetic patient. For the non-diabetic group, survival increased only slightly when CHX was used (94.3 %, 5.7 % failures), compared to when CHX was not used (91.8 %, 8.2 % failures) [22, 26].

## Conclusions

The literature included to this review is very heterogeneous concerning the investigated objects, methods, and conclusions. Diabetes is a group of metabolic diseases in which there are high blood sugar levels over a prolonged period. When looking at the complications and side effects resulting from diabetes, it is important to know which type of diabetes the patient suffers from, if there is any therapy, which kind of therapy, the grade of glycemic control, and duration of the disease. The previously described micro- and macroangiopathies develop with the duration and repetitions of elevated glycemic periods. In most studies, these information are missing and there is only a dichotomy classification of diabetes or healthy. Most studies include patients with well-controlled diabetes, and there is no or little effect on implant survival. Many authors conclude that prospective long-time studies are needed to answer the issues. On the other hand, it would be non-ethical to observe patients with poor glycemic control, because health-threatening systemic side effects developed.

Analyzing the available studies, we conclude as follows:

Dental implants are safe and predictable procedures for dental rehabilitation in diabetics. The survival rate of implants in diabetics does not differ from the survival rate in healthy patients within the first 6 years, but in the long-term observation up to 20 years, a reduced implant survival can be found in diabetic patients. Patients with poorly controlled diabetes seem to have delayed osseointegration following implantation. After 1 year, there is no difference between diabetic and healthy individuals, not even to the poorly controlled HbA1c. Therefore, we recommend avoiding immediate loading of the implants. In the first years after implant insertion, there seems to be no elevated risk of peri-implantitis; but in the long-term observation, peri-implant inflammation seems to be increased in diabetic patients. Therefore, a risk-adapted dental recall is helpful to detect early signs of gingivitis, which can easily be treated by dental/implant cleanings to avoid serious peri-implant infection. We found some hints that good glycemic control improves osseointegration and implant survival. Therefore, and to avoid other long-term side effects, the practitioner should ask for the HbA1c and if necessary improvement of antidiabetic therapy should be aimed. In the literature, we found no evidence that bone augmentation procedures like guided bone regeneration and sinus lifts have a higher complication and failure rate in patients with well- to fairly well-controlled diabetes. To improve implant survival and reduce postoperative complications, supportive therapy consisting of prophylactic antibiotics and chlorhexidine mouth rinse is recommended.
